# The complete mitochondrial genome of Florida gar (*Lepisosteus platyrhincus*)

**DOI:** 10.1080/23802359.2016.1144092

**Published:** 2016-03-28

**Authors:** Hui Yu, Jia Li, Zhiqiang Ruan, Xingyu Ma, Jimin Zhang, Min Wang, Qiong Shi, Xinxin You

**Affiliations:** a BGI Education Center, University of Chinese Academy of Sciences, Shenzhen, China;; b Shenzhen Key Lab of Marine Genomics, Guangdong Provincial Key Lab of Molecular Breeding in Marine Economic Animals, BGI, Shenzhen, China;; c BGI-Zhenjiang Detection Co., LTD, BGI-Zhenjiang Institute of Hydrobiology, Zhenjiang, China

**Keywords:** *Lepisosteus platyrhincus*, mitogenome, phylogenetic analysis

## Abstract

*Lepisosteus platyrhincus* is a member of the family Lepisosteidae living in the Western Hemisphere. It is a primitive air-breathing fish with the special intermediate position of phylogeny and between elasmobranchs and teleosts. Herein, we first sequenced and assembled the complete mitochondrial genome of *Lepisosteus platyrhincus*. The total length of mitochondrion is 16 320 bp with GC content of 42.43%, containing 13 protein-coding genes, two ribosomal RNA (rRNA) genes, 22 transfer RNA (tRNA) genes and a 564 bp control region. The accuracy of the fresh sequences was verified by phylogenetic analysis. This mitochondrial genome provides potentially important resources for addressing taxonomic issues and studying molecular evolution.

The Florida gar, *Lepisosteus platyrhincus*, a primitive air-breathing fish, is a member of the family Lepisosteidae. The gars are non-teleost bony fishes that share the common ancestry with *Amia*, belonging to the order Semionotiformes (Frick et al. 2007). As an ancient group, it extends back to about 180 million years ago; hence, this species appeared earlier than most teleosts (Orlando et al. 2003; Orlando et al. 2007). To date, the study about *L. platyrhincus* is very few. We collected the sample in Guangzhou, China (E 113°17', N 23°8') and extracted genomic DNA from muscular tissue of *L. platyrhincus* using phenol–chloroform method (Taggart et al. 1992). The specimen was stored at China National Genebank (accession no. GZ2014122012). Then we sequenced the complete mitochondrial genome using Illumine Hiseq 4000 platform (Illumina, San Diego, CA) at BGI-Shenzhen, China and assembled it with the *SOAPdenovo-Trans* (Kmer-71) (Tang et al. 2015). All experiments followed the guidelines of the Animal Ethics and were approved by the Institutional Review Board on Bioethics and Biosafety of BGI.

The complete mitochondrial genome sequence of *L. platyrhincus* (Genbank accession no. KU199002) is 16 320 bp long with the majority of mitogenome characteristic structure that includes 13 protein-coding genes, two rRNA genes (12S rRNA and 16S rRNA), 22 tRNA genes and a control region (D-loop) which is located between *tRNA-Pro* and *tRNA-Phe*. They are annotated by the online service Dogma (Wyman et al. 2004). Most of the mitochondrial genes are located on the heavy strand, while *ND6* and eight tRNA genes (*tRNA-Gln*, *tRNA-Ala*, *tRNA-Asn*, *tRNA-Cys*, *tRNA-Tyr*, *tRNA-Ser*, *tRNA-Glu* and *Trna-Pro*) lie on the light strand. In descending order, the total base composition is A (30.97%), C (27.18%), T (26.60%) and G (15.26%). Similar to *L. oculatus* (NC_004744.1), the GC content is 42.43%. Interestingly, all the protein-coding genes use ATG as start codon except *COX1* with GTG and *ND6* with GGT. The length of every tRAN gene varies from 64 bp (*tRNA-Cys*) to 73 bp (*tRNA-Leu*). Besides, two rRNA genes are annotated. They are 953 bp (12S rRNA) and 1 598 bp (16S rRNA) in length.

Moreover, to further verify the reliability of the newly acquired sequence and its annotation, we downloaded complete mitochondrial genome sequences of other 9 closely related species from NCBI for phylogenetic analysis. Muscle Program (vision: v3.8.31) (Edgar 2004) was used to execute the whole mitochondrial genome alignment. Subsequently, we chose the conserved blocks by the Gblocks (http://phylogeny.lirmm.fr/). A neighbour-joining tree (maximum-likelihood) was constructed based on the conserved blocks by using MEGA 6.0 Program (Tamura et al. 2013). According to the established phylogenetic tree, we confirm that the Florida gar is much closer to the spotted gar (see more details in Figure 1), which coincides to the real situation.

**Figure 1.**
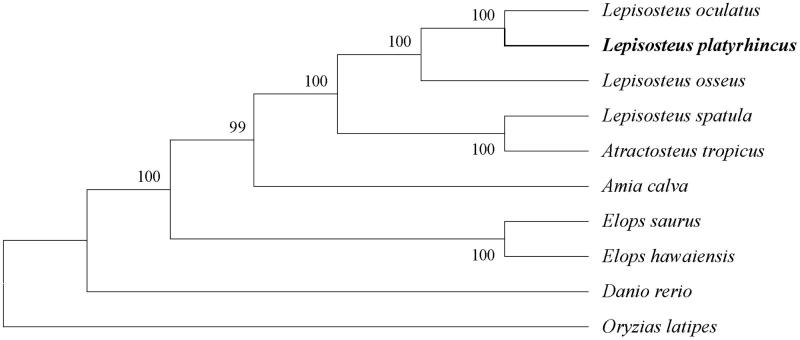
The rectangular phylogenetic tree based on the conserved blocks of ten related mitochondrial genomes. Accession nos. *Lepisosteus oculatus*, NC_004744.1; *Lepisosteus osseus*, NC_008104.1; *Lepisosteus spatula*, NC_008131.1; *Atractosteus tropicus*, NC_024178.1; *Amia calva*, NC_004742.1; *Elops saurus*, NC_005803.1; *Elops hawaiensis*, NC_005798.1; *Danio rerio*, NC_002333.2; *Oryzias latipes*, NC_004387.1.
